# BmNPV p35 Reduces the Accumulation of Virus-Derived siRNAs and Hinders the Function of siRNAs to Facilitate Viral Infection

**DOI:** 10.3389/fimmu.2022.845268

**Published:** 2022-02-18

**Authors:** Shudi Zhao, Guanping Chen, Xiangshuo Kong, Nan Chen, Xiaofeng Wu

**Affiliations:** ^1^ College of Animal Sciences, Zhejiang University, Hangzhou, China; ^2^ Key Laboratory of Silkworm and Bee Resource Utilization and Innovation of Zhejiang Province, Hangzhou, China

**Keywords:** insect immunity, RNAi, noncoding RNAs, baculovirus, virus–insect interaction

## Abstract

Antiviral immunity involves various mechanisms and responses, including the RNA interference (RNAi) pathway. During long-term coevolution, viruses have gained the ability to evade this defense by encoding viral suppressors of RNAi (VSRs). It was reported that *p35* of baculovirus can inhibit cellular small interference RNA (siRNA) pathway; however, the molecular mechanisms underlying *p35* as a VSR remain largely unclear. Here, we showed that *p35* of *Bombyx mori* nucleopolyhedrovirus (BmNPV) reduces the accumulation of virus-derived siRNAs (vsiRNAs) mapped to a particular region in the viral genome, leading to an increased expression of the essential genes in this region, and revealed that *p35* disrupts the function of siRNAs by preventing them from loading into Argonaute-2 (Ago2). This repressive effect on the cellular siRNA pathway enhances the replication of BmNPV. Thus, our findings illustrate for the first time the inhibitory mechanism of a baculovirus VSR and how this effect influences viral infection.

## Introduction

All living organisms are constantly exposed to all sorts of microbial pathogens. The conflict between viral pathogens and their hosts leads to the coevolution of antiviral defenses and viral suppression mechanisms of host resistance. As for insects, the RNA interference (RNAi) pathway is an important immune strategy against viruses. It has been validated that both RNA viruses such as *Drosophila* X virus (DXV) (1) and cricket paralysis virus (CrPV) (2) and DNA viruses such as invertebrate iridescent virus 6 (IIV6) (3) are targets of insect RNAi machinery. In antiviral RNAi response of insects, Dicer-2 (Dcr-2) and Argonaute-2 (Ago2) are two key proteins. Double-stranded RNAs (dsRNAs) produced in viral infection are detected and processed into approximately 20 nucleotides (nt) virus-derived siRNAs (vsiRNAs) by the nuclease Dcr-2 (4). Following dsRNA cleavage, the generated siRNAs are loaded into Ago2 to form the RNA-induced silencing complex (RISC), which induces the antiviral defense machinery to cleave specific complementary viral RNAs with the RNase activity of Ago2 (5, 6).

Baculoviruses are a large group of entomopathogenic viruses with double-stranded DNA genomes of 80–180 kb contained in rod-shaped nucleocapsids (7). Due to their high virulence and rapid establishment of infection, baculoviruses are widely used for biological pest control and exploited as vector for heterologous protein expression (8, 9). As a representative member of baculoviruses, *Bombyx mori* nucleopolyhedrovirus (BmNPV) is the main pathogen of silkworm, an important economic insect for silk production (10, 11), and causes great economic losses to sericulture around the world (12). Although DNA viruses do not replicate through a dsRNA intermediate, overlapping transcripts produced by compact genomes lead to significant amounts of dsRNA (13). Consistently, vsiRNAs can be detected during infections with baculoviruses and other insect DNA viruses (3, 14–16), and viral infections are improved in RNAi-defective mutants for these cases (17–20).

As an inevitable result of coevolution and natural selection, it is a universal phenomenon that viruses have developed countermeasures against the antiviral RNAi response of insects (21, 22). They encode viral suppressors of RNAi (VSRs) to inhibit the host RNAi pathway by diverse molecular mechanisms. For example, the protein 1A of *Drosophila* C virus (DCV) binds with long dsRNAs and shield them from Dcr-2-mediated cleavage (2). The protein B2 encoded by Flock House virus (FHV) binds with both dsRNAs and siRNAs to hinder their participation in the RNAi pathway (23–25) and also interacts with Dcr-2 to reduce siRNA production (26). Additionally, 1A of CrPV blocks catalytic activity of the RISC by direct interaction with Ago2 (27, 28). VP1 of Nora virus inhibits the cleavage ability of preassembled mature RISC by antagonizing Ago2 slicer activity (29, 30). Apart from VSRs encoded by RNA viruses, 340R of IIV6 stabilizes long dsRNAs and binds with siRNAs to inhibit its loading into RISC (31, 32). Also, it has been reported that *p35* of baculovirus, which is well known as an inhibitor of apoptosis (33), has the ability to suppress cellular RNAi pathway in diverse insect and mammalian cells (34). However, the effect on viral infection and the molecular mechanisms underlying *p35* as a VSR remain largely unknown. Considering the antiviral immunity of the RNAi pathway and the application of baculovirus, addressing these questions could not only bring a better understanding on the mechanisms of baculovirus infection and the detail of baculovirus-host interaction but also provide theoretical guidance for the prevention of the disease caused by baculovirus or for baculovirus-mediated lepidoptera pest control.

In this study, by using small RNA sequencing (RNA-seq), we found that p35 reduces the amount of vsiRNA derived from the region located in 50k–95k nucleotides (nt) of the minus strand of BmNPV genome, in which the essential genes of BmNPV are densely distributed. As predicted, the expression of most essential genes in this region increased due to the reduced vsiRNA level. Moreover, we found that p35 can prevent siRNA from being loaded into Ago2, which blocks the downstream steps of cellular RNAi pathway. As a result, weakened antiviral RNAi promotes the proliferation of BmNPV in the presence of p35, and such effect is independent of its inhibitory function of apoptosis. Taken together, our results indicated the dual role of p35 in evading antiviral RNAi and promoting viral infection, namely, changing the population of vsiRNAs and inhibiting their downstream functions.

## Materials and Methods

### Cell Line, Viruses, and Antibodies

The BmN cell line was cultured at 27°C in SF900 II SFM (Gibco) supplemented with 3% fetal bovine serum (FBS; Gibco). The T3 strain of BmNPV was maintained in our laboratory and used as the wild-type (WT) virus. The antibodies used in this study including anti-GP64 (Sigma, A2980), anti-GFP (TransGene, HT801), anti-Flag (HuaBio, 0912-1), anti-HA (Yeasen, 30702ES60), and anti-α-tubulin (Beyotime, AF5012) were commercially available.

### Expression Plasmids and Transfection

The expression plasmids used in this study including pIZ-EGFP, pIZ-p35, pIZ-p35shift, pIZ-p35Flag, and pIZ-BmAgo2-HA were generated from the pIZ/V5-His plasmid. The pIZ/V5-His was preserved in our laboratory, and its multiple cloning sites (MCSs) had been optimized to be the same as the MCS of pFastBacHTB plasmid. All insertion open reading frames (ORFs) of the plasmids were confirmed by sequencing in both directions.

For transfection of the expression plasmids, BmN cells were resuspended and equally added to individual wells of a 6-well plate. Once monolayers had formed, the medium was replaced by 1ml TC-100 medium (Sangon, E60032) before transfection medium consisting of 125 μl TC-100 medium, 5 μl Lipo8000 (Beyotime, C0533), and 3 μg plasmid was added. The cells were incubated for 5 h after addition of the mixture and then replenished with 1.5 ml of SF900 medium supplemented with 3% FBS. For transfection of BmNPV bacmid, the method was similar. In brief, the medium of BmN cells was replaced, and a mixture containing 250 μl TC-100 medium, 8 μl LipoInsect (Beyotime, C0551), and 16 μg bacmid was added. The medium was rechanged after 4 h.

### Generation of the p35KO and p35V71P Mutants of BmNPV

To generate the p35KO mutant, a *p35* knockout BmNPV bacmid was constructed *via* λ-Red homologous recombination as described previously (35–37). A linear chloramphenicol acetyltransferase (*cat*) gene cassette flanked by 50-nt fragments homologous with the adjacent regions of the *p35* ORF that mediate the insertion of the *cat* gene into the *p35* locus while concomitantly deleting the *p35* ORF (**Figure S1A**) was amplified from plasmid pKD3. The PCR fragment was gel purified and electroporated into *Escherichia coli* BW25113 competent cells containing the BmNPV bacmid and the temperature-sensitive plasmid pKD46 (38). Transformants were screened with kanamycin and chloramphenicol and confirmed by PCR (**Figures S1B**, **E–H**). For visualization of the viral infection, polyhedrin (polh) of BmNPV with its own promoter was inserted into the *polh* locus of the resulting *p35*KO bacmid using pFastBacDual plasmid *via* Tn7-mediated transposition, as previously described (39–41) (**Figure S1A**).

As for the p35V71P mutant, the mutated *p35* gene whose nucleotides coding for the valine 71 were changed to those for proline was amplified *via* overlap extension PCR (**Figure S1C**), and mutation was confirmed by sequencing in both directions (**Figure S1D**). This mutated gene was then inserted into the *polh* locus together with the *polh* gene *via* Tn7-transpositon to generate the p35V71P bacmid (**Figure S1A**). The two mutant viruses were amplified and purified from BmN cells transfected with the corresponding bacmids.

### Gene Silencing

To silence the genes, we used RNAi by generating dsRNA synthesized *in vitro* using the T7 RNAi transcription kit (Vazyme, TR102) according to the instructions from the manufacturer. T7 promoter sequences were incorporated in both forward and reverse primers designed to amplify the DNA fragments of target genes as transcription templates for dsRNA. The control template in this kit was used to generate dsCtr as a negative control.

For transfection of the dsRNA, BmN cells were resuspended and equally added to individual wells of a 6-well plate. Once monolayers had formed, the medium was replaced by 1ml TC-100 medium before transfection medium consisting of 125 μl TC-100 medium, 5 μl LipoRNAi (Beyotime, C0535), and 5 μg dsRNA was added. The cells were incubated for 5 h after addition of the mixture and then replenished with 1.5 ml of SF900 medium supplemented with 3% FBS.

### Quantitative Real-Time PCR Analysis

BmN cells (1 × 10^6^) infected with indicated viruses at a multiplicity of infection (MOI) of 10 or transfected with dsRNAs were harvested at the given time points. Total RNA of the cells was extracted using RNAiso Plus (Takara, 9109) to synthesize the first-strand cDNAs with the PrimeScript™ RT reagent Kit (Takara, RR047). Reactions were performed using Hieff^®^ qPCR SYBR Green Master Mix (Yeasen, 11203) and run on a Light Cycler 480 (Roche). *Bmrpl32* (Gene ID 778453) was used for normalizing the data. Three technical and three biological replicates per treatment were analyzed using the 2^-△△Ct^ method to calculate the relative expression levels of selected genes. Student’s two-tailed t test was used for significant difference analysis between two samples. The statistical analysis and figure generation were completed by GraphPad Prism 8.

### Analysis of Viral Growth Curve and Genome Copies

Viral growth curve analysis was conducted as described previously (42). In brief, BmN cells were infected in triplicate with indicated viruses at an MOI of 10. After 1 h of incubation at 27°C, fresh medium was added to cells, and this time point was defined as 0 h post infection (hpi). At the given time points, cells were centrifuged and supernatants containing the BV were harvested. The titers of BV were determined by TCID_50_ end-point dilution assay in BmN cells (43).

To quantify viral genomic DNA (gDNA) copies, total DNA of sediments obtained from the centrifugation was extracted using Universal Genomic DNA Extraction Kit (Takara, 9765). Viral gDNA copies were determined by quantification of a viral gene *gp41* (Gene ID 1488698) by qPCR. DNA concentrations were measured by a NanoDrop instrument (Thermo), and 30 ng total genomic DNA was used for each qPCR reaction. *Bmrpl27* (Gene ID 692703) served as an internal normalization control, and the 0 h sample was used as input to normalize the data. The differences in means between two or three sets of data at a certain time point were compared by t test or one-way ANOVA. GraphPad Prism 8 was used for statistical analysis and figure generation.

### Western Blot

BmN cells (1 × 10^6^) of different treatment conditions were harvested at the designated time points and lysed in cell lysis buffer (Beyotime, P0013) for 30 min on ice. Protein concentrations in lysates were determined using Bicinchoninic Acid (BCA) Protein Assay Kit (Takara, T9300A). For each sample, 20 μg of protein extract was separated by a 12% Sodium Dodecyl Sulfate Polyacrylamide Gel Electrophoresis (SDS PAGE) gel and transferred onto a polyvinylidene fluoride (PVDF) membrane. The membrane was blocked with 5% non-fat milk in Tris Buffered Saline with Tween (TBST) for 2 h and incubated with corresponding antibodies for 1.5 h at room temperature or overnight at 4°C. Thereafter, Horseradish Peroxidase (HRP)-conjugated antibodies were used for enhanced chemiluminescence. α-Tubulin served as an internal reference.

### Small RNA Sequencing and Analyses

BmN cells (1 × 10^7^) were infected with BmNPV WT or p35KO mutant virus at an MOI of 10. After 48 h of infection, cells were harvested and total RNA of the cells was extracted. The small RNAs were size selected by PAGE gel, and the small RNAs ranging from 15 to 35 nt were excised. Approximately 3 μg of RNA per sample was used as the input material for library preparation, the libraries were generated using NEB Next Multiplex Small RNA Library Prep Set for Illumina (NEB), and index codes were added to attribute sequences to each sample. The prepared libraries were sequenced on an Illumina Hiseq 2500/2000 platform (Allwegene), and 50-bp single-end reads were generated.

For the analysis of viral small RNAs, raw reads generated from 3 independent libraries were cleaned using Trim Galore (Version 0.6.6) and mapped to the BmNPV genome (GenBank accession NC_001962) using Bowtie2 (Version 2.4.2) (44). Mapped reads were normalized to library size and expressed as reads per million (RPM). For the genome distribution, the number of 5’ ends of all vsiRNAs at each position was plotted. miRNA levels were analyzed by mapping the cleaned reads to the *B. mori* genome (SilkBase) (45). The union of differentially expressed miRNAs between two groups was used for miRNA heat map. Differential expression analysis of two groups was performed using R package edgeR (Version 3.30.3), and P value of 0.05 was set as the threshold for significant differential expression. Figures were generated using R package ggplot2 (Version 3.3.2) or pheatmap (Version 1.0.12) and embellished using Adobe Illustrator.

### Apoptosis Detection of BmN Cells

BmN cells were resuspended and equally added to individual wells of a 96-well plate. At designated time points, BmNPV p35KO or p35V71P mutant was added at an MOI of 10. Apoptosis degree of the cells was determined by the activity of caspase-3 and caspase-7 using Caspase-Glo 3/7 Assay System (Promega, G8090) (46). Caspase-Glo 3/7 substrate and buffer in the kit were mixed, and each well was added by 100 μl of the reagent simultaneously. The luminescent signal was measured by Microplate Reader (Bio Tek) after incubation for 1 h. Caspase 3/7 activity was represented by relative light unit (RLU). Three replications were set for each time point, and the fresh medium served as the blank control.

### β-Elimination

BmN cells (1 × 10^6^) transfected with siRNA of Enhanced Green Fluorescent Protein (siEGFP) for 48 h were harvested, and small RNA of the cells was extracted using RNAiso for Small RNA (Takara, 9753). β-Elimination of the RNA was performed as the protocol described before (47).

In this study, 40 µg of RNA in 47.5-µl nuclease-free water was oxidized by addition of 12.5 µl 200 mM NaIO_4_ and 40 µl 5× borate buffer and incubation at room temperature for 30 min. In the control group, NaIO_4_ was replaced by RNase-free water. Unreacted NaIO_4_ was quenched by addition of 10 µl glycerol and incubation at room temperature for 10 min. To induce β-elimination, 10 µl of 500 mM NaOH was added and the reaction was kept at 45°C for 90 min. The treated RNA was purified by ethanol precipitation.

### Northern Blot

Northern blot analysis was performed as the protocol described before (48). Here, 10 μg of small RNA were separated on a 7-M urea-denaturing 15% polyacrylamide gel and transferred onto a nylon membrane. siRNA Ladder Marker (TaKaRa, 3430) was used as a molecular size marker. UV-cross-linked membrane was prehybridized for 3 h and hybridized with biotin-labeled DNA probe. Viral probe was synthesized using Biotin Random Prime DNA Labeling Kit (Beyotime, D3118). The signal was detected using Chemiluminescent Biotin-labeled Nucleic Acid Detection Kit (Beyotime, D3308). Ethidium bromide–stained 5S rRNA served as an internal reference.

### Cell Viability Assay

Cell viability was determined using Cell Counting Kit-8 (Beyotime, C0038) and Calcein-AM/PI Live-Dead Cell Staining Kit (Solarbio, CA1630) according to the protocol provided by the manufacturers. In brief, BmN cells in 96-well plate were infected with BmNPV at an MOI of 10 at designed time points. And the cells were added with corresponding reagent. The absorbance of each well was measured at a test wavelength of 450 nm.

### Co-Immunoprecipitation and RNA Immunoprecipitation

For RNA immunoprecipitation, BmN cells (1 × 10^6^) co-transfected with siEGFP and pIZ-BmAgo2-HA for 48 h were harvested and washed twice by PBS and then lysed using the lysis buffer with freshly added 1 mM Dithiothreitol (DTT), 200 U/ml RNase inhibitor (Promega), and protease inhibitor cocktail (Bimake, B14001) for 30 min at 4°C. Here, 10% of lysates were used as protein inputs and 20% of lysates were used for small RNA isolation as RNA inputs. The remnant lysates were prepared for immunoprecipitation overnight at 4°C using anti-HA magnetic beads (Bimake, B26201). Immunoprecipitation beads were washed with lysis buffer five times. Then, 25% of the washed beads were used for Western blotting, and the remnant beads were used for RNA extraction.

The co-immunoprecipitation is similar. In brief, cells cotransfected with pIZ-p35Flag and pIZ-BmAgo2-HA were lysed using the lysis buffer with freshly added cocktail for 30 min at 4°C. Here, 20% of lysates were used as inputs and remnant lysates were used for immunoprecipitation overnight at 4°C using Anti-Flag magnetic beads (Bimake, B26101). Immunoprecipitation beads were washed with PBS five times and used for Western blotting.

## Results

### BmNPV Is a Target of Cellular SiRNA Pathway

Baculovirus-derived siRNAs from *Helicoverpa armigera* single nucleopolyhedrovirus (HaSNPV)-infected HzFB cells and *Autographa californica* multiple nucleopolyhedrovirus (AcMNPV)-infected Sf9 cells have been reported previously (14, 34). Nonuniformly distributed vsiRNAs on viral genomes resulted in hot spots with a large number of mapped vsiRNAs and cold spot with a low number of mapped vsiRNAs. Also, the expression of genes in hot spots instead of cold spots was affected when Dcr-2 was silenced (18). To confirm the significance of cellular siRNA pathway in resistance of BmNPV, the key factors, Dcr-2 and Ago2, were knocked down respectively *via* dsRNAs, and the silencing efficacy was examined by qPCR. After 48 h of dsRNA transfection, the gene-silenced cells were infected with BmNPV, and the effect on viral amplification was assessed by quantifying viral titers and gDNA copies at various time points as well as the expression level of GP64, a key envelope protein of BmNPV.

**Figure 1:**
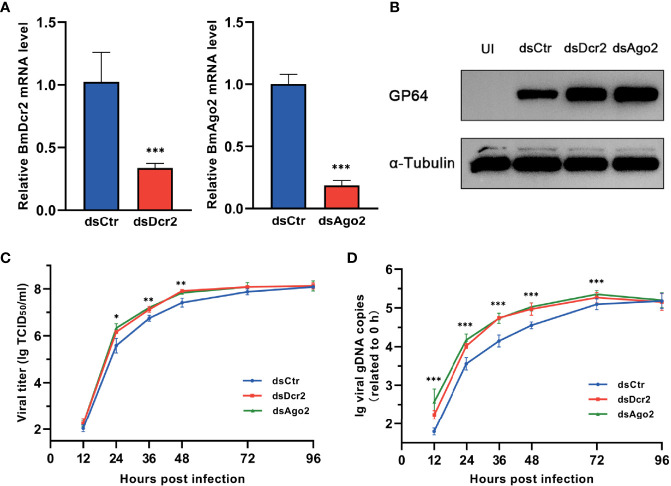
BmNPV infection is restricted by cellular RNAi pathway. **(A)** qRT-PCR confirmed the decline of Dcr-2 and Ago2 expression in BmN cells treated with dsDcr2 and dsAgo2 at 48 h post transfection relative to dsCtr-transfected control group. **(B)** Western blot analysis of viral envelope protein GP64 expression at 48 hpi. α-Tubulin was used as an internal reference. **(C)** Viral growth curves determined by TCID_50_ endpoint dilution assays in dsDcr2- and dsAgo2-treated cells and the dsCtr-treated control group. (log-transformed data). **(D)** Replication kinetics of BmNPV in BmN cells under the indicated treatments. qPCR data of viral gDNA copies were normalized to housekeeping gene *Bmrpl27* and presented relative to the 0 hpi time point. (log-transformed data) (*P < 0.05, **P < 0.01, ***P < 0.001). dsCtr, control dsRNA; dsDcr-2, dsRNA for Dcr-2; UI, uninfected; dsAgo2, dsRNA for Ago2.

After 48 h of dsRNA transfection, the expression of Dcr-2 and Ago2 was dramatically decreased (**Figure 1A**). As a consequence, the expression of GP64 was enhanced (**Figure 1B**), and viral replication was also increased. During 12–48 h, viral titers in Dcr-2- and Ago2-silenced groups showed a modest but significant increase compared with the negative control (**Figure 1C**). Also, viral gDNA copy number in RNAi-defective groups was significantly higher than the control until 96 h postinfection (**Figure 1D**). These results illustrated that BmNPV is also a target of RNAi like the viruses mentioned above, and its infection progress can be restricted by the siRNA pathway of the host. However, the siRNA pathway seems to make no difference on viral invasion at a relatively late stage, and it may be due to the overwhelming viral load that is unaffordable for the RNAi-mediated antiviral immunity.

### p35 of BmNPV Restricts SiRNA Pathway of the Host

**Figure 2:**
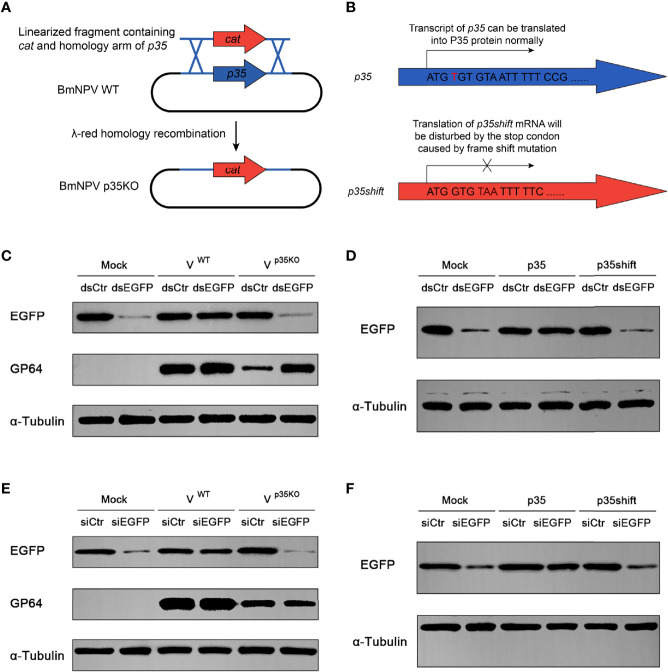
Cellular RNAi pathway is restricted by BmNPV p35. **(A)** Strategy to generate the *p35*-defective BmNPV mutant (p35KO). *p35* was replaced by a chloramphenicol resistance gene *cat* through λ-red homologous recombination. **(B)** Strategy to construct the negative control of p35 expression plasmid (p35shift). A stop codon was introduced next to the start codon that disables the translation of p35. **(C–F)** RNAi efficiency represented by Western blot analysis of EGFP expression in BmN cells treated by dsRNA and WT or p35KO BmNPV **(C)**, dsRNA and p35 or p35shift plasmid **(D)**, siRNA and WT or p35KO BmNPV **(E)**, siRNA and p35 or p35shift plasmid **(F)**. GP64 was used to represent the existence of BmNPV, and α-tubulin served as an internal reference.

In a previous study, *p35* of AcMNPV was found to be responsible for the suppression of RNAi in diverse insect and mammalian cells, while the molecular mechanism of this suppression was not elucidated (34). Before exploring this mechanism, we firstly verified the RNAi suppression effect of BmNPV *p35*. To visualize the RNAi efficiency, the constructed pIZ-EGFP plasmid was cotransfected with dsRNA of EGFP (dsEGFP) into BmN cells. Subsequently, the cells were infected with wild type and p35-deleted BmNPV (**Figure 2A**). After 24 h of infection, the cells were harvested to detect the expression level of EGFP. In cells treated by dsEGFP without the existence of p35, namely, mock and p35KO virus-infected cells, the expression level of EGFP was much lower than that in the dsCtr-treated cells, while no obvious difference was observed between the dsCtr and dsEGFP treatments of WT virus-infected cells (**Figure 2C**). These results indicated that the p35 gene of BmNPV was responsible for the suppression of cellular siRNA pathway.

To investigate whether the suppression effect is independent of viral infection, we constructed a pIZ-p35 plasmid to express p35 of BmNPV *in vitro* and a negative control pIZ-p35shift, in which a stop codon was introduced after the p35 ATG start codon by frameshift mutation to exclude the influence of p35 transcript (**Figure 2B**). As expected, after 48 h of cotransfection, the nullification of dsEGFP was only observed in pIZ-p35-transfected cells (**Figure 2D**), indicating that it is an individual function of p35.

### p35 Mainly Interferes With Steps Downstream of Dcr-2 Cleavage in Gene Silencing

In siRNA pathway, there are two major parts for gene silencing, namely, Dcr-2 cleavage and its downstream steps. Considering that p35 is able to restrict cellular siRNA pathway, we wonder which step is primarily responsible for this effect. In contrast to dsRNA, transfected siRNAs do not require processing by Dcr-2 and directly participate in the next steps. Therefore, it is suitable to estimate the relative importance of Dcr-2 cleavage and its downstream steps by detecting siRNA-induced gene silencing efficiency of EGFP. If inhibition of Dcr-2 function is the predominant mechanism, p35 will fail to restrict the function of directly introduced siRNA, and *vice versa*.

In siEGFP-transfected cells, the expression of EGFP was obviously inhibited. Such an inhibitory effect could also be observed in p35KO BmNPV-infected and pIZ-p35shift-transfected BmN cells, but not in WT BmNPV or pIZ-p35-treated cells (**Figures 2E, F**). These results indicated that the skip of dsRNA cleavage cannot avoid the suppression of siRNA pathway, and the steps downstream of Dcr-2 cleavage are primarily responsible for this p35-mediated suppression.

### p35 Impedes Virus-Derived Small Interference RNA From Loading on Argonaute-2

Previous results showed that the silencing efficiency of introduced siRNA could also be effectively restricted by p35 (**Figures 2E, F**), implying that the steps downstream of Dcr-2-induced dsRNA slicing were affected. After being processed by Dcr-2, resulted siRNAs must be loaded onto Ago2 to execute their function (6, 49, 50). Therefore, we hypothesized that this key process of RISC formation was blocked by p35 therein. To directly test this hypothesis, we analyzed sensitivity to β-elimination of introduced siEGFP. In previous studies, once siRNAs were incorporated in Ago2, a 2′-O-methyl group would be added on their 3′ termini by the protein Hen1 (51–53). Such a methylation modification endows siRNAs with the resistance to β-elimination. Unmodified siRNAs are sensitive to the reaction, being converted into RNAs with 3′-monophosphate by the reaction. The resulting RNAs migrate faster in polyacrylamide gel electrophoresis (47, 54), making it visible to determine whether the siRNA is loaded onto Ago2.

**Figure 3:**
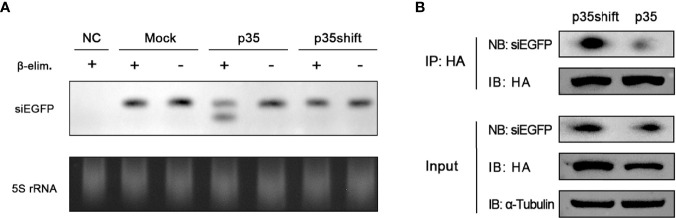
p35 prevents siRNA from loading onto Ago2. **(A)** Northern blot analysis of transfected siEGFP in BmN cells transfected with pIZ-p35 or pIZ-p35shift expression plasmid, and the samples were subjected to β-elimination (+) or mock treated (−). Cells without siEGFP transfection were used as the negative control (NC). Ethidium bromide–stained 5S rRNA was used as a loading control. **(B)** RNA immunoprecipitation assay of transfected pIZ-BmAgo2-HA and siEGFP in BmN cells transfected with pIZ-p35shift or pIZ-p35 plasmid. (IB, immunoblotting; NB, Northern blotting).

We conducted Northern blot to detect the effect of the β-elimination reactions and found that in the pIZ-p35-transfected cells, an appreciable fraction of siEGFP was sensitive to β-elimination, as evident from the increased mobility on gel, indicating that it had not been loaded into Ago2. In contrast, transfected siEGFP in mock-transfected and pIZ-p35shift-transfected cells was not affected by β-elimination, indicating that they were incorporated with Ago2 (**Figure 3A**).

To further confirm that p35 prevents siRNA from loading on Ago2, we performed RNA immunoprecipitation assay with anti-HA magnetic beads by using pIZ-BmAgo2-HA- and siEGFP-transfected BmN cells in the absence or presence of p35. As expected, the binding between Ago2 and siRNA was significantly suppressed (**Figure 3B**). Together, these results proved that p35 can obstruct the steps downstream of Dcr-2 cleavage by preventing siRNAs from associating with RISC.

### p35 Reduces the Accumulation of Virus-Derived Small Interference RNAs Derived From Certain Regions of Viral Genome

**Figure 4:**
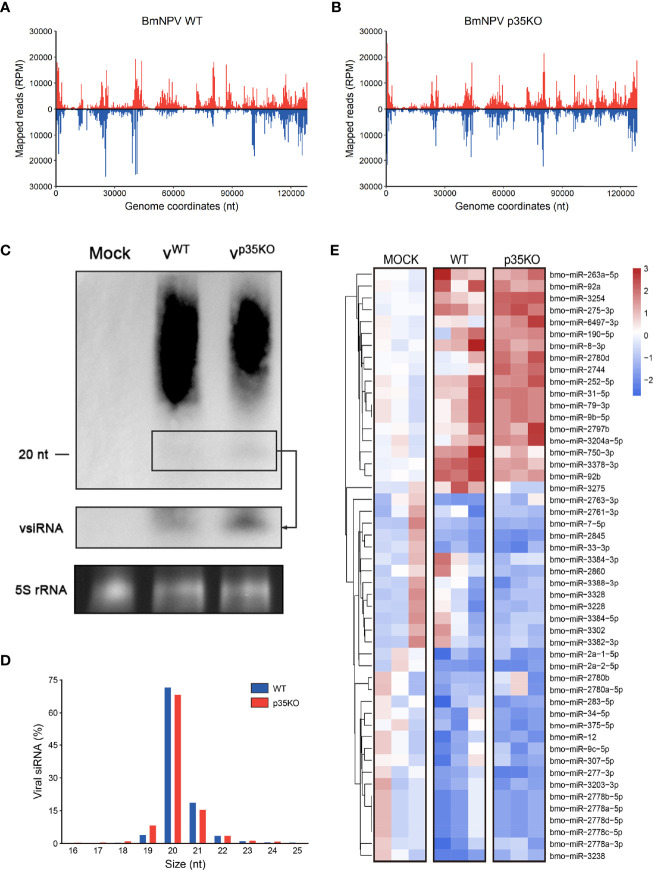
p35-induced alternation in vsiRNA accumulation and miRNA expression. **(A, B)** Distribution of vsiRNAs across the viral genome after infection of WT **(A)** and p35KO **(B)** BmNPV, with vsiRNAs mapping to the plus and minus strands of the genome in red and blue, respectively. The average counts (n = 3) of all vsiRNAs at each position were plotted at 400 nt resolution. **(C)** Northern blot analysis of small RNA derived from the 50k–95k nt in the BmNPV genome coordinates at 48 hpi. A longer exposure of the blot in the black box allows visualization of the vsiRNAs. Ethidium bromide–stained 5S rRNA was used as a loading control. **(D)** Size profile of vsiRNAs from WT and p35KO BmNPV-infected BmN cells. Size distribution as the average percentage (n = 3) of total vsiRNAs is presented. **(E)** Heatmap of the changes in miRNA abundance in WT and p35KO BmNPV-infected BmN cells. Three libraries are presented separately. Hierarchical clustering is based on the averages of the 3 libraries. Color coding indicates log2-transformed fold changes relative to mock infection. (50 differentially expressed miRNAs with higher significance were selected to show).

The functional mechanisms of VSRs are diverse. Some of them can restrict both Dcr-2 cleavage to alter vsiRNA generation and the formation of RISC to block the function of siRNA, as is described in the *Introduction*. Although p35 mainly affects the downstream steps of siRNA pathway, we also would like to make clear its role in vsiRNA accumulation. To this end, small RNA-seq of BmN cells infected with WT or p35KO BmNPV was conducted and the small RNAs mapped to BmNPV genome were analyzed. As observed before (14), small RNAs in both treatments were predominantly 20 nt in length (**Figure 4D**), which coincides with the typical size of Dcr-2 products. However, to our surprise, the distribution of mapped vsiRNAs across the viral genome was not similar in WT and p35KO BmNPV infection. Instead, in the context of WT BmNPV infection, the count of vsiRNAs mapped to approximately 50k–95k nt was evidently lower than that in the context of p35KO BmNPV infection (**Figures 4A, B**). Correspondingly, most of the genes in this region were mapped to more vsiRNAs in the absence of p35 (**Figure S3A**). To validate this phenomenon, Northern blot analysis was performed and the probes were generated from the viral fragments in the genome coordinates of 50k–95k nt. Consistent with the sequencing result, the band of approximately 20-nt vsiRNA complementary with probes appears more pronounced in the p35KO BmNPV-infected cells (**Figures 4C** and **S3B**), indicating a disequilibrium induced by BmNPV p35 in the accumulation of vsiRNA.

In plants, Ago1 is a shared factor of antiviral RNAi and miRNA pathways that can load with both vsiRNAs and miRNAs (55). Due to this convergence of functions, VSRs of plants can affect cellular miRNAs (56). In contrast, Dicer and Argonaute proteins are exclusive in miRNA and siRNA pathways of insects (57). We thus analyzed miRNA profiles of uninfected and WT or p35KO BmNPV-infected cells to evaluate the role of p35 in miRNA pathway. The length distribution was not affected during viral infection, and most of the detected miRNAs were 20–22 nt as expected (**Figure S3C**). In both virus-infected cells, a reduction in the level was observed for the majority of changed cellular miRNAs, while the difference between the two virus-infected libraries was not remarkable. Generally, there appeared to be an identical trend of miRNAs in the infection of WT and p35KO BmNPV. However, two small sets of miRNAs were worthy of attention. One was represented by miR-2780d and miR-2744, which were only induced by p35KO BmNPV, and another was represented by miR-3275, which was only upregulated by p35 therein and might be related to some biological functions of p35 (**Figures 4E** and **S3D**). Together, these data suggest that p35 has little effect on the expression of cellular miRNA and the length distribution of small RNAs but alters the vsiRNA accumulation in the viral genome coordinates of 50k–95k nt.

### The Decline of Virus-Derived Small Interference RNA in the 50k–95k Region Improves the Expression of Viral Genes in This Region

**Figure 5:**
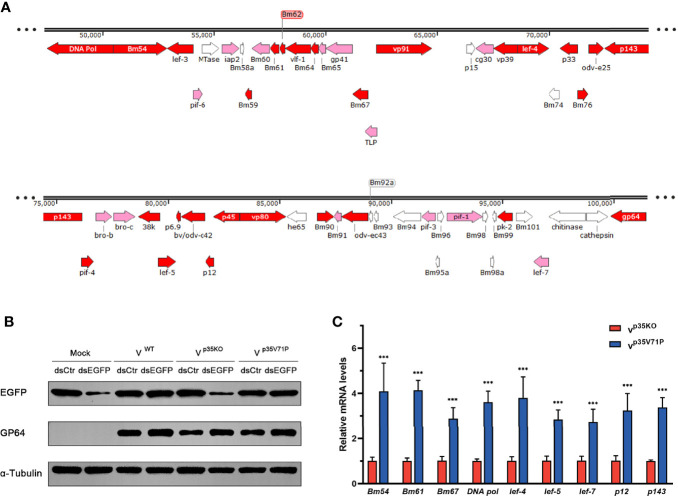
p35-induced vsiRNA decline promotes expression of genes in this region. **(A)** Distribution of genes in the 50k–95k region of the BmNPV genome with essential genes and important genes in red and pink, respectively. **(B)** Inhibition effect on siRNA pathway induced by the indicated viruses. GP64 was used to represent the existence of BmNPV, and α-tubulin served as an internal reference. **(C)** qRT-PCR analysis for the expression of genes in the 50k–95k region of viral genome at 48 hpi. ***P < 0.001.

Since the existence of p35 reduced the vsiRNA accumulation of the 50k–95k nt in the BmNPV genome coordinates, we focused on the specific property of this region. To this end, we analyzed the distribution of genes important for viral replication and spread and found that the distribution density of essential genes (virus lacking these genes will not be able to survive) and important genes (viral infection and spread will be severely restricted without these genes) in the 50k–95k region was higher than that of other regions in the BmNPV genome (**Figure 5A**) (7, 58). Therefore, we examined how the decrease of vsiRNA affects the expression of these genes in the 50k–95k region. Considering that p35 is also well known as an inhibitor of apoptosis besides its VSR function (33), to eliminate the influence of apoptosis induced by p35KO BmNPV infection, we mutated valine 71 of BmNPV p35 to proline (p35V71P), which was previously shown to disrupt the spatial conformation of the reactive loop structure in p35 and to completely abolish its antiapoptotic activity by failing to inhibit the caspase activity (59, 60). The mutated p35 gene was confirmed by sequencing and recombined into p35KO BmNPV to generate the p35V71P-mutated virus.

The functional effect of BmNPV p35V71P was examined first. As expected, after destruction of the crucial site of apoptosis resistance, the mutated virus failed to restrict cellular caspase activity as the WT BmNPV did (**Figure S2C**), and apoptosis induced by p35V71P BmNPV in BmN cells was as severe as that in p35KO BmNPV-infected cells (**Figure S4**). Nevertheless, the function of p35V71P as a VSR was not affected by this single amino acid mutation. In p35V71P mutated virus-infected cells, gene silencing mediated by dsEGFP was obviously inhibited, and the inhibition effect was the same as the WT BmNPV (**Figure 5B**), demonstrating it an ideal strategy to avoid the discrepancy produced by distinct apoptosis rate with no impact on the suppression of cellular RNAi. Eight essential genes and 1 important gene (lef-7) in 50k–95k region were selected to analyze whether the altered vsiRNA level affects the gene expression of this region. In the p35V71P-infected BmN cells, mRNA levels of the tested genes showed an increase of 3~4-fold (**Figure 5C**). Taken together, our results indicated that the p35-induced decline of vsiRNA mapped to the 50k–95k of the BmNPV genome coordinates promoted the expression of critical genes in this region, which may create a favorable condition for viral infection.

### The Restriction of RNAi Induced by p35 Facilitates Viral Infection

We have observed an enhanced viral infection in cells with RNAi defects caused by Dcr-2 or Ago2 knockdown and an increased gene expression in the region with reduced vsiRNA yield caused by the existence of BmNPV p35. Therefore, we hypothesized that the p35-mediated inhibitory effect on host siRNA pathway will contribute to an improved viral infection. In order to avoid the diversity in the level of cell apoptosis, we also used the mutant virus p35V71P as the control of p35KO mutant virus.

**Figure 6:**
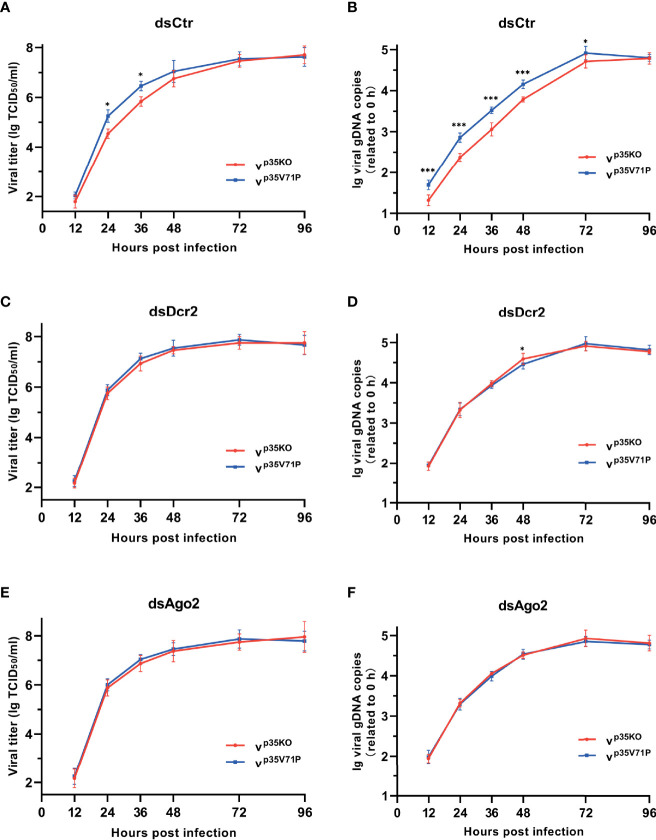
p35-induced suppression on cellular RNAi facilitates viral infection. **(A, C, E)** Viral growth curves of p35KO and p35V71P BmNPV mutants determined by TCID_50_ endpoint dilution assays in dsCtr **(A)**-, dsDcr2 **(C)**-, and dsAgo2 **(E)**-treated cells. (log-transformed data). **(B, D, F)** Replication kinetics of the two mutated viruses under the indicated treatments. qPCR data of viral gDNA copies were normalized to housekeeping gene *Bmrpl27* and presented relative to the 0 hpi time point. (log-transformed data) (*P < 0.05, ***P < 0.001).

To assess the impact of the impaired siRNA pathway caused by p35, we infected BmN cells with the two mutant viruses respectively and monitored viral titers and DNA levels over time. A consistent infection and replication defect were observed in p35KO virus, with 4.2–5.1-fold lower viral titers and 2.4–3.3-fold lower DNA levels compared with the control (**Figures 6A, B**). To vindicate that such differences was due to the p35-induced inhibition on cellular RNAi instead of other variables, we generated RNAi-defective cells by knocking down the key factors of siRNA pathway, Dcr-2 or Ago2 as mentioned above, and infected these cells with the two mutant viruses, respectively. As expected, the differences in viral titers and DNA levels were not observed. Mutant viruses p35KO and p35V71P replicated with similar kinetics and accumulated to similar levels in RNAi mutant cells (**Figures 6C–F**). It is because that p35-induced suppression cannot lead to discrepancies in these RNAi-impaired cells. However, it is noteworthy that in the case of viral infection, cellular siRNA pathway was not completely blocked by p35, based on the fact that in the presence of p35, RNAi defects caused by Dcr-2 or Ago2 knockdown still led to significantly enhanced viral infection and accumulation (**Figure S5**). Overall, these data indicated that the VSR function of BmNPV p35 can create a favorable condition for the establishment of viral infection and the replication of viral genome during a relatively early stage, even though the antiviral activity of cellular siRNA pathway cannot be fully masked.

## Discussion

Unlike vertebrates, insects lack acquired immunity against specific viruses, while the RNAi pathway allows them to resist viral infection pointedly by capturing intermediates derived in viral metabolism and therefore plays a vital role in antiviral immunity. Facilitated by their large coding capacity and high mutation rate, both DNA viruses and RNA viruses have evolved various antagonists or modulators of diverse immune pathways including RNAi pathway during the long-term arms race (61–63). Here, we use an RNAi suppressor-defective mutant of BmNPV and combine small RNA-req with biochemistry experiments to show that p35 of BmNPV antagonizes cellular RNAi pathway through two ways, namely, reducing the accumulation of vsiRNAs mapped to the genome region where critical genes of virus are densely distributed and hindering vsiRNA from loading on Ago2 to block the downstream function of antiviral RNAi reaction. Such suppression on cellular RNAi enhances the expression of the critical genes and promotes the infection of BmNPV.

VSRs have been identified in over 10 insect-specific viruses. However, due to the difficulty of the experimental system to manipulate viral genomes of interest, these proteins are often characterized in isolation of transgenic expression, using recombinant proteins, or in heterologous systems (27, 31, 64, 65). By generating RNAi suppressor-defective virus, p35KO, the vsiRNA map of BmNPV-infected cells was sketched and it reveals for the first time the alteration on vsiRNA accumulation across the viral genome in the absence of p35. In addition, though the antiviral response toward baculoviruses and the VSR function of p35 have been reported before (14, 18, 34), its mechanism remained unclear. Our current data of the dual mechanism of p35-induced RNAi suppression and its influence on viral gene expression and infection may fill these gaps left by previous studies.

With respect to RNAi against dsDNA viruses, an overlap of transcripts of adjacent genes on opposite strands and the presence of secondary structures in transcripts are assumed to be the reason for the production of dsRNAs (13, 66, 67). Our results have shown in this study that p35 reduces the accumulation of vsiRNAs derived from the 50k–95k region to promote the expression of genes in this region. We supposed that the generation pathway may be affected by p35 through some mechanisms, such as temporally and spatially regulating the ratio of overlapping transcripts on opposite strands to reduce the probability of dsRNA formation, altering the stability of newly formed double-strand intermediates, disguising virus-derived transcripts as endogenous products, or preventing the Dcr-2 recognition of certain dsRNAs. The uneven distribution of vsiRNA mapped to baculovirus genome has already been reported (7, 18), while it was vague about the reason for this nonuniform distribution. We are also curious about why p35 mainly affects the level of vsiRNA in the genome coordinates of 50k–95k, which is coincidently the region with high density of crucial genes for replication and dispersal, and the mechanisms for this preference on the alternation of vsiRNA distribution remain to be further investigated. In addition to the impact on siRNA accumulation, we also observed that BmNPV modifies the expression of two small sets of cellular miRNAs in a p35-dependent manner, though the impact on other miRNA trends is relatively slight (**Figures 4E** and **S3D**), as is mentioned in *p35 Reduces the Accumulation of Virus-Derived Small Interference RNAs Derived From Certain Regions of Viral Genome*. Viral manipulation of the cellular miRNA expression is a common phenomenon for DNA viruses (68, 69), and further investigation on the mechanism and function of this impact will lead to a better understanding on the role of p35 in the small RNA field.

It should be noted that though the p35-induced antagonism on host RNAi evidently facilitates viral replication and gene expression, the antiviral activity of this immune pathway cannot be completely eliminated by p35 (**Figures S5C, D**). However, when monitoring the inhibition efficiency on RNAi using EGFP reporter gene, we found that the interference effect on EGFP expression almost completely disappeared in the presence of p35 (**Figures 2C–F**). Baculoviruses are highly pathogenic to insects with the ability to rapidly replicate and spread (70). Much more amount of siRNA will be produced in viral infection compared with the condition of transfection. A high level of vsiRNA may fully occupy the VSR function of p35, leading to the incomplete inhibition on host RNAi pathway, while the RNAi response triggered by transfected dsEGFP or siEGFP is finite, which can be effectively masked by p35. Previous results may support this hypothesis that a very large amount of dsRNA (80–160 μg) was also able to induce gene silencing in the presence of baculovirus (71, 72), which was much more than that used in this study (5 μg) for an equal number of cells in the absence of virus, and overexpression of p35 enhanced viral replication through suppression of antiviral RNAi response (18), implying that the VSR function of p35 was saturated.

Previous study has shown that p35 blocks RNAi downstream pathway instead of blocking dsRNA cleavage, which was concluded from the RNAi response activated by transfected dsRNA (7). By using a p35KO BmNPV mutant, our current data show that p35 can also influence the upstream of RNAi response activated by viral infection, while it is consistent with the earlier view that p35 suppresses the gene silencing mainly in the downstream RNAi pathway. We also directly confirmed for the first time that p35 hinders the Ago2 loading to disable the subsequent functions of siRNAs. However, no obvious interaction was observed between p35 and Ago2 (**Figure S2D**), implying that another mechanism is adopted in this suppression, perhaps by interacting with siRNAs to sequester them from Ago2, indirectly antagonizing the RNA binding activity of Ago2, or interfering with the function of other factors involved in this step. Further investigations on these questions are still needed and will shed more light on the understanding of viral strategies to optimize the cellular environment for viral life cycle and provide new insights into the viral interaction with the host antiviral innate immune response.

## Data Availability Statement

The datasets presented in this study can be found in online repositories. The names of the repository/repositories and accession number(s) can be found below: https://www.ncbi.nlm.nih.gov/, PRJNA792944.

## Author Contributions

XW conceived and designed the experiments and revised the article. SZ designed this study, performed the experiments, processed the data, and wrote the article. GC, XK, and NC helped with the experiments. All authors contributed to the article and approved the submitted version.

## Funding

This work was supported by the National Natural Science Foundation of China (31972619/32172793) and the Natural Science Foundation of Zhejiang Province (Z20C170008).

## Conflict of Interest

The authors declare that the research was conducted in the absence of any commercial or financial relationships that could be construed as a potential conflict of interest.

## Publisher’s Note

All claims expressed in this article are solely those of the authors and do not necessarily represent those of their affiliated organizations, or those of the publisher, the editors and the reviewers. Any product that may be evaluated in this article, or claim that may be made by its manufacturer, is not guaranteed or endorsed by the publisher.

## Supplementary Material

Supplementary Figure 1 Construction of recombinant bacmids. **(A)** Strategy for construction of p35KO and p35V71P bacmids. The ORF of *p35* was replaced by a chloramphenicol acetyltransferase gene (*cat*) *via* λ-red homologous recombination. The indicated fragments were inserted into the *polh* locus to generate BmNPV p35KO and p35V71P mutants. **(B)** Diagram indicating the relative positions of primers A, B, C, and D (arrows) used for PCR screening of *p35* deletion. The expected sizes of PCR products amplified from specific primer pairs were indicated. **(C)** Primers to mutate the nucleotides in *p35* gene coding for the valine 71 into those for proline. **(D)** Sequencing confirmation of the mutation indicated in **(C)**. **(E–H)** The results of agarose gel electrophoresis showing PCR products generated from primer pairs of A-B **(E)**, A-D **(F)**, C-B **(G)** and C-D **(H)** using *p35* knockout or WT bacmid as template. **(I–J)** The results of agarose gel electrophoresis showing semi-quantitative RT-PCR products generated from primer pairs of qp35-F and qp35-R, and the cDNA from WT or p35KO BmNPV infected cells **(I)** and pIZ-p35shift + p35KO virus or pIZ-p35 + p35KO virus treated cells **(J)** was used as template.

Supplementary Figure 2 Cell viability after viral infection and the interaction between Ago2 and p35. **(A)** Cell Survival State determined using the Calcein-AM/PI staining. almost all of the cells are alive before 48 hpi (showed by AM staining), and only a small minority of the cells died at 72 and 96 hpi (showed by PI staining). **(B)** Cell viability determined using CCK-8 assay. **(C)** Apoptosis analysis of BmN cells infected with WT, p35KO and p35V71P BmNPV. Apoptosis degree was determined by Caspase activity and showed by luminescent intensity. **(D)** No significant interaction between BmAgo2 and p35 was observed in Co-IP assay.

Supplementary Figure 3 p35-induced alternation on small RNAs. **(A)** Volcano plot of genes mapped to different amount of vsiRNA in p35KO and WT viruses infected cell samples. Red plots represent genes mapped to up-regulated vsiRNA amount in p35KO sample and blue plots represent genes mapped to down-regulated vsiRNA amount, black plots represent genes with no significant changed vsiRNA amount. The significant standard is p_value<0.05, |FoldChange| ≥ 0.5. Annotated genes are those in the 50k-95k region of viral genome. **(B)** Northern blot analysis of small RNA derived from the 50k-95k nt in the BmNPV genome coordinates. The numbers represent relative signal intensity ratios of p35KO vs WT, and the data was generated by ImageJ and normalized using 5S rRNA. (complete picture of , including 3 replications and the first replication was showed in ). **(C)** Size profile of miRNAs from WT, p35KO BmNPV-infected and mock-infected BmN cells. Size distribution as the average percentage (n=3) of total miRNAs is presented. **(D)** Heatmap of the abundance of all the differentially expressed miRNAs in WT and p35KO BmNPV-infected BmN cells compared with the mock group.

Supplementary Figure 4 Apoptosis degree of BmN cells after infection of p35KO or p35V71P mutated viruses at indicated time points. Uninfected cells and WT BmNPV-infected cells at 96 hpi. were served as control.

Supplementary Figure 5 p35 fails to completely mask the antiviral activity of RNAi. The same data was presented in , but now grouped by viral genotype. **(A, C)** Viral growth curves of p35KO (A) and p35V71P (C) BmNPV mutants determined by TCID_50_ endpoint dilution assays with indicated treatments. (log-transformed data). **(B, D)** Replication kinetics of the two mutated viruses under the indicated treatments. qPCR data of viral gDNA copies was normalized to housekeeping gene *Bmrpl27* and presented relative to the 0 hpi time point. (log-transformed data) (*P < 0.05, **P < 0.01, ***P < 0.001).
