# Supplementary figures and images for: BmNPV p35 Reduces the Accumulation of Virus-Derived siRNAs and Hinders the Function of siRNAs to Facilitate Viral Infection
Source: Front Immunol. 2022 Feb 18;13:845268. doi: 10.3389/fimmu.2022.845268 (PMC8895250; doi:10.3389/fimmu.2022.845268)

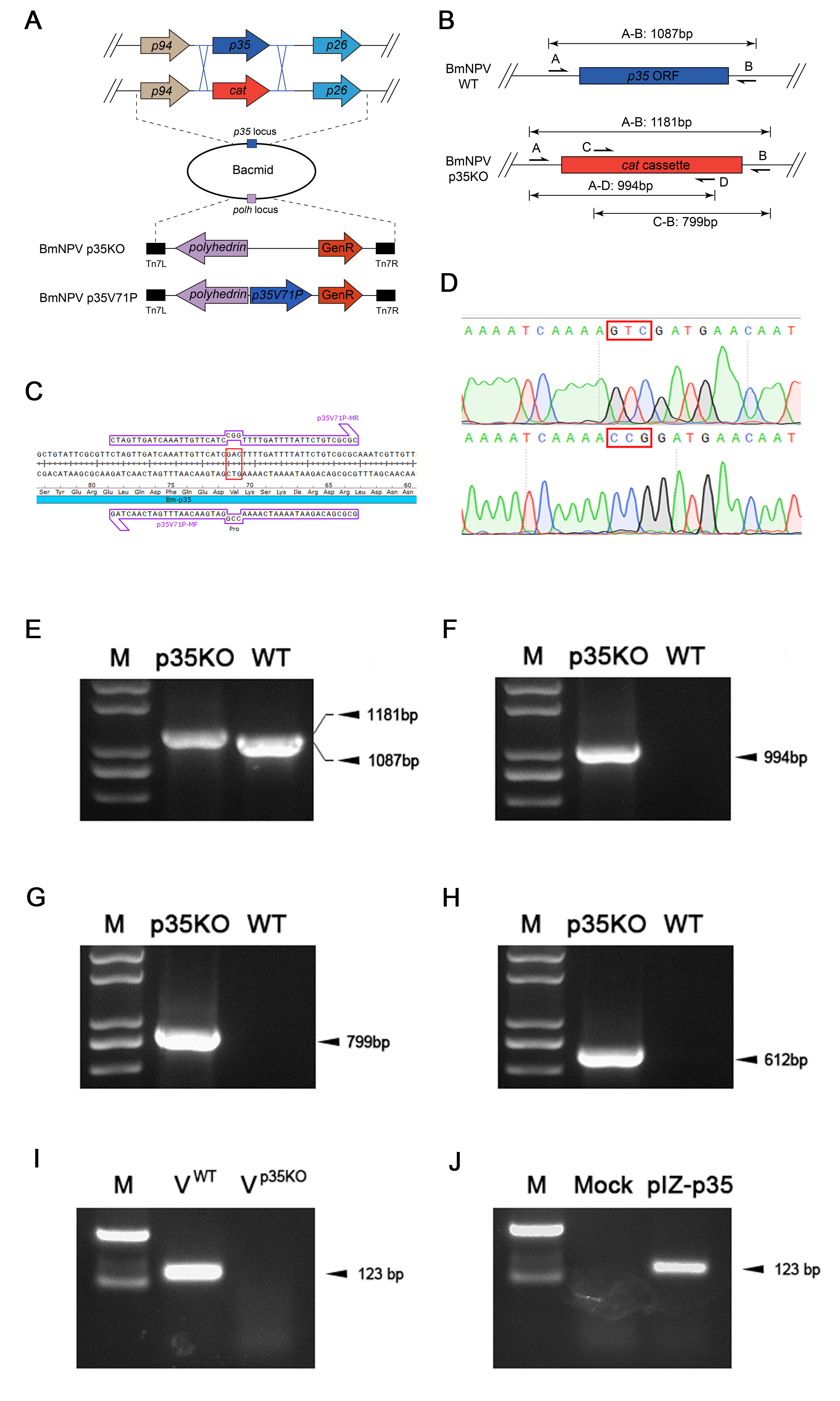

Supplement: Supplementary Figure 1 — Construction of recombinant bacmids. (A) Strategy for construction of p35KO and p35V71P bacmids. The ORF of p35 was replaced by a chloramphenicol acetyltransferase gene (cat) via λ-red homologous recombination. The indicated fragments were inserted into the polh locus to generate BmNPV p35KO and p35V71P mutants. (B) Diagram indicating the relative positions of primers A, B, C, and D (arrows) used for PCR screening of p35 deletion. The expected sizes of PCR products amplified from specific primer pairs were indicated. (C) Primers to mutate the nucleotides in p35 gene coding for the valine 71 into those for proline. (D) Sequencing confirmation of the mutation indicated in (C). (E–H) The results of agarose gel electrophoresis showing PCR products generated from primer pairs of A-B (E), A-D (F), C-B (G) and C-D (H) using p35 knockout or WT bacmid as template. (I–J) The results of agarose gel electrophoresis showing semi-quantitative RT-PCR products generated from primer pairs of qp35-F and qp35-R, and the cDNA from WT or p35KO BmNPV infected cells (I) and pIZ-p35shift + p35KO virus or pIZ-p35 + p35KO virus treated cells (J) was used as template. [file Image_1.jpeg]

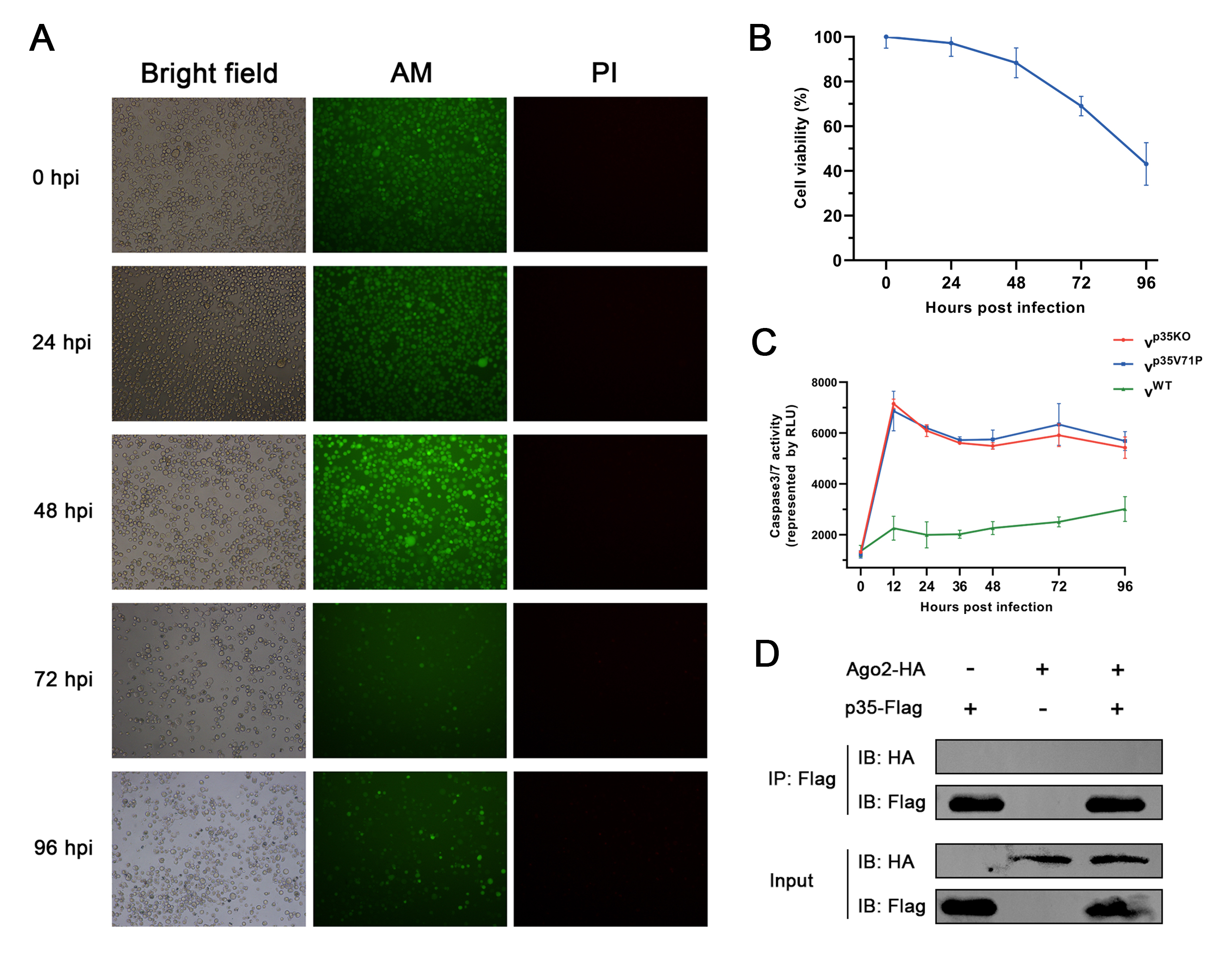

Supplement: Supplementary Figure 2 — Cell viability after viral infection and the interaction between Ago2 and p35. (A) Cell Survival State determined using the Calcein-AM/PI staining. almost all of the cells are alive before 48 hpi (showed by AM staining), and only a small minority of the cells died at 72 and 96 hpi (showed by PI staining). (B) Cell viability determined using CCK-8 assay. (C) Apoptosis analysis of BmN cells infected with WT, p35KO and p35V71P BmNPV. Apoptosis degree was determined by Caspase activity and showed by luminescent intensity. (D) No significant interaction between BmAgo2 and p35 was observed in Co-IP assay. [file Image_2.jpeg]

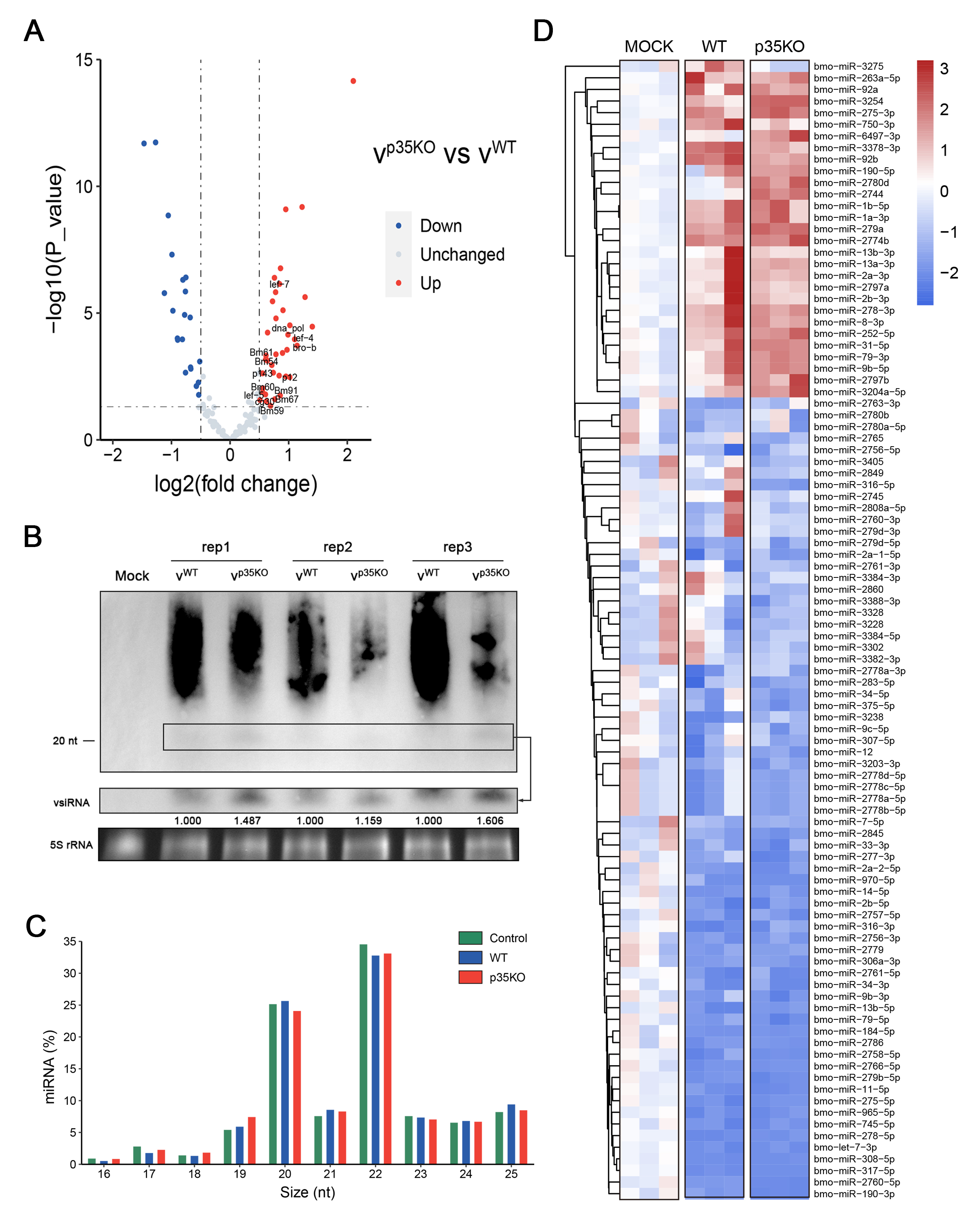

Supplement: Supplementary Figure 3 — p35-induced alternation on small RNAs. (A) Volcano plot of genes mapped to different amount of vsiRNA in p35KO and WT viruses infected cell samples. Red plots represent genes mapped to up-regulated vsiRNA amount in p35KO sample and blue plots represent genes mapped to down-regulated vsiRNA amount, black plots represent genes with no significant changed vsiRNA amount. The significant standard is p_value<0.05, |FoldChange| ≥ 0.5. Annotated genes are those in the 50k-95k region of viral genome. (B) Northern blot analysis of small RNA derived from the 50k-95k nt in the BmNPV genome coordinates. The numbers represent relative signal intensity ratios of p35KO vs WT, and the data was generated by ImageJ and normalized using 5S rRNA. (complete picture of , including 3 replications and the first replication was showed in ). (C) Size profile of miRNAs from WT, p35KO BmNPV-infected and mock-infected BmN cells. Size distribution as the average percentage (n=3) of total miRNAs is presented. (D) Heatmap of the abundance of all the differentially expressed miRNAs in WT and p35KO BmNPV-infected BmN cells compared with the mock group. [file Image_3.jpeg]

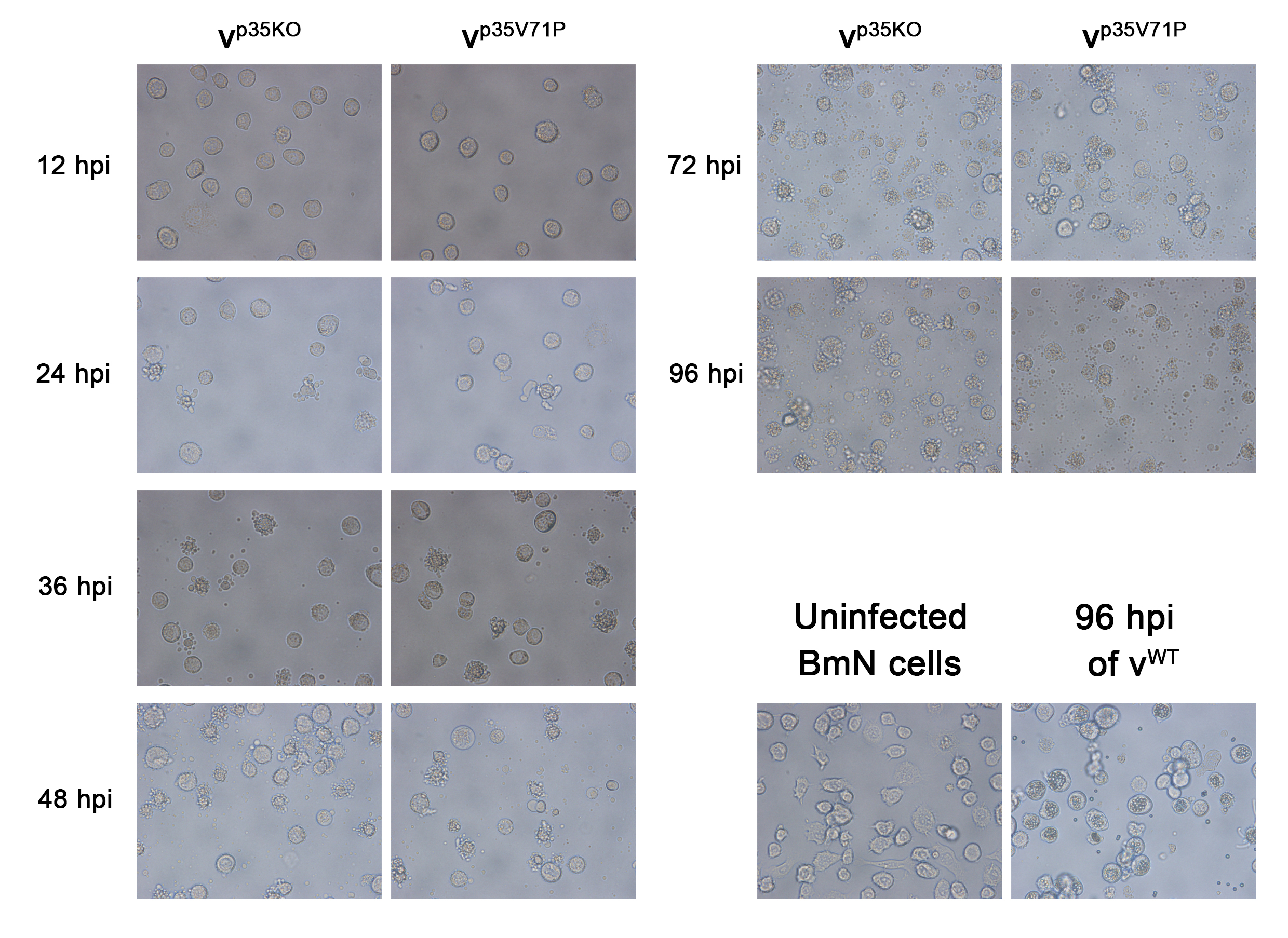

Supplement: Supplementary Figure 4 — Apoptosis degree of BmN cells after infection of p35KO or p35V71P mutated viruses at indicated time points. Uninfected cells and WT BmNPV-infected cells at 96 hpi. were served as control. [file Image_4.jpeg]

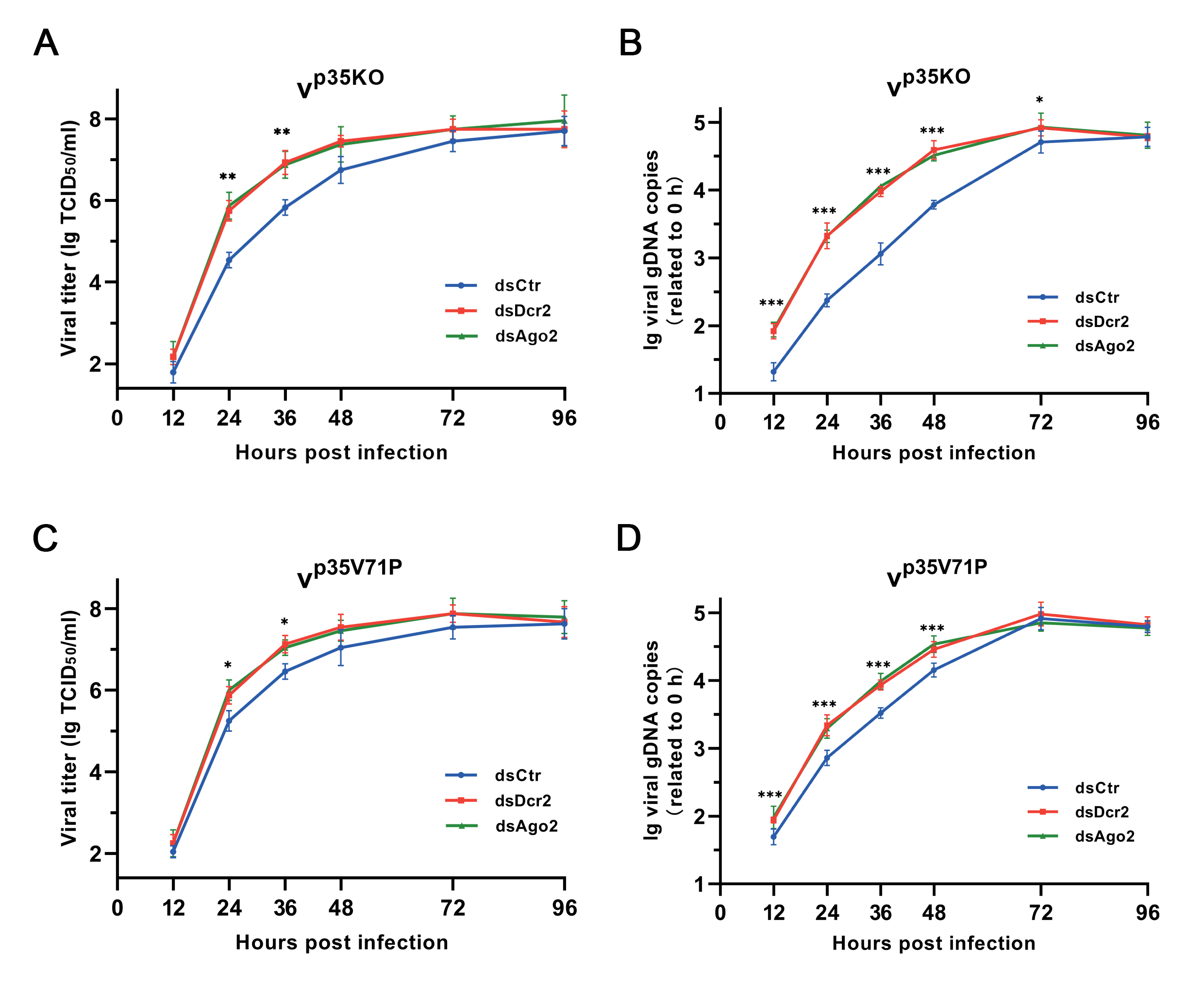

Supplement: Supplementary Figure 5 — p35 fails to completely mask the antiviral activity of RNAi. The same data was presented in , but now grouped by viral genotype. (A, C) Viral growth curves of p35KO (A) and p35V71P (C) BmNPV mutants determined by TCID50 endpoint dilution assays with indicated treatments. (log-transformed data). (B, D) Replication kinetics of the two mutated viruses under the indicated treatments. qPCR data of viral gDNA copies was normalized to housekeeping gene Bmrpl27 and presented relative to the 0 hpi time point. (log-transformed data) (*P < 0.05, **P < 0.01, ***P < 0.001). [file Image_5.jpeg]
