# Supplementary material for: BmNPV p35 Reduces the Accumulation of Virus-Derived siRNAs and Hinders the Function of siRNAs to Facilitate Viral Infection
Source: Front Immunol. 2022 Feb 18;13:845268. doi: 10.3389/fimmu.2022.845268 (PMC8895250; doi:10.3389/fimmu.2022.845268)
Supplement: Supplementary file 6 [file Table_1.docx]

**Supplementary Tables**

**Supplementary Table 1.** Primers, probes and siRNAs used in this study

| **Primers** | | |
| --- | --- | --- |
| Name | Sequences (5′-3′) | Applications |
| dsDcr2_T7-F | GGATCCTAATACGACTCACTATAGGGACTACAACAGAAGGAATACAG | Amplification of the template for dsDcr2 (T7 promoter sequence is underlined) |
| dsDcr2-R | ACTCTCATTGGATGGTCTTC |  |
| dsDcr2-F | GACTACAACAGAAGGAATACAG |  |
| dsDcr2_T7-R | GGATCCTAATACGACTCACTATAGGACTCTCATTGGATGGTCTTC |  |
| qDcr2-F | TGAATTGGCACGAAACCAGG | qRT-PCR analysis of BmDcr2 |
| qDcr2-R | TGTCGCCGTTAAACCTAGAACT |  |
| dsAgo2_T7-F | GGATCCTAATACGACTCACTATAGGCCTTCTTCTGAAAGTCAGCCTTCAG | Amplification of the template for dsAgo2 (T7 promoter sequence is underlined) |
| dsAgo2-R | GATGGAGCTTTTGAATATTTGATGT |  |
| dsAgo2-F | CCTTCTTCTGAAAGTCAGCCTTCAG |  |
| dsAgo2_T7-R | GGATCCTAATACGACTCACTATAGGGATGGAGCTTTTGAATATTTGATGT |  |
| qAgo2-F | GCTCCTAAAAGTGAAGAGTCC | qRT-PCR analysis of *BmAgo2* |
| qAgo2-R | GGTCCTGGAATTGCAGGTTC |  |
| dsEGFP_T7-F | GGATCCTAATACGACTCACTATAGGGAGGGCGATGCCACCTACG | Amplification of the template for dsEGFP (T7 promoter sequence is underlined) |
| dsEGFP-R | TCAGGGCGGACTGGGTGC |  |
| dsEGFP-F | GAGGGCGATGCCACCTACG |  |
| dsEGFP_T7-R | GGATCCTAATACGACTCACTATAGGGTCAGGGCGGACTGGGTGC |  |
| pIZ-EGFP-F | GCTCTAGAATGGTGAGCAAGGGC | Construction of pIZ-EGFP (XbaI and HindIII restriction site are underlined) |
| pIZ-EGFP-R | GCCAAGCTTTTACTTGTACAGCTCG |  |
| pIZ-p35-F | CGGGATCCATGTGTGTAATTTTTC | Construction of pIZ-p35 and pIZ-p35shift (BamHI and HindIII restriction site are underlined) |
| pIZ-p35shift-F | CGGGATCCATGGTGTAATTTTTCC |  |
| pIZ-p35-R | GCCAAGCTTTTATTTAATCATGTC |  |
| pFB-polh-F | GCGTATACTTAACGATACAAATGGAAATA | Rescue of polh and p35V71P for the generation of the p35KO and p35V71P mutants (BstZ17I, HindIII and NheI restriction site are underlined. Nucleotides for amino acid mutation are in bold) |
| pFB-polh-R | GCAAGCTTTTAATACGCCGGACCAGTGAA |  |
| p35V71P-F | GCGTATACGGTCACGTTCTGTAACATGAG |  |
| p35V71P-MR | CTAGTTGATCAAATTGTTCATC**CGG**TTTTGATTTTATTCTGTCGCGC |  |
| p35V71P-MF | GCGCGACAGAATAAAATCAAAA**CCG**GATGAACAATTTGATCAACTAG |  |
| p35V71P-R | GCGCTAGCTTATTTAATCATGTCTAATATTACATTTTTGTT |  |
| p35KO-F | TCAACGTTGCTTGTATTAAGTGAGCATTTGAGCTTTACCATTGCAAATAACATATGAATATCCTCCTTAG | Amplification of linear CAT cassette with homologous arms (homologous arms are underlined) |
| p35KO-R | ACACAATCAAGCAATGACAAAGAATAATATTAGGCAATAAATTTTAACATGTGTAGGCTGGAGCTGCTTC |  |
| p35KO-A | GAGCTTTACCGAACGGTTAT | Confirming the deletion of p35 ORF |
| p35KO -B | CAAATCTTGCGTTACGAGTAG |  |
| p35KO -C | CGTTGATATATCCCAATGGC |  |
| p35KO -D | ACTCATCGCAGTACTGTTGT |  |
| qp35-F | CAAACACAACGACTGCTACG | Confirmation of p35 knockout and overexpression |
| qp35-R | TGAGCAAACGGCACAATAAC |  |
| BmAgo2-HA-R | CTA**AGCGTAATCTGGTACGTCGTATGGGTA**GACGAAGAACATACGGCTCTGTTTCAGCA | Construction of pIZ-BmAgo2-HA and pIZ-p35-Flag (pIZ-BmAgo2-HA was constructed using Gibson assembly, homologous arms and HindIII restriction site are underlined, HA and Flag tags are in bold). The gene id of BmAgo2 is 692544 |
| pIZ-BmAgo2-F | ACGAGCTCACTAGTCGCGGCCGCTTTCGAAATGGCTAGAGGAAAAAACAAAGGTGGTAA |  |
| pIZ-HA-R | GCTGATTATGATCCTCTAGTACTTCTCGACCTA**AGCGTAATCTGGTACGTCGTATGGGT** |  |
| pIZ-vector-F | GTCGAGAAGTACTAGAGGAT |  |
| pIZ-vector-R | TTCGAAAGCGGCCGCGACTA |  |
| pIZ-p35Flag-R | GCCAAGCTTTTA**CTTATCGTCGTCATCCTTGTAATC**TTTAATCATGTC |  |
| qBm54-F | GTATGAGCACGCTTTGCGAG | qRT-PCR analysis of *Bm54* of BmNPV |
| qBm54-R | TGATATCTCGGCCGGCAAAA |  |
| qBm61-F | ACCGAACTGGTCAAATCGACA | qRT-PCR analysis of *Bm61* of BmNPV |
| qBm61-R | TAGACTTGTTCGCACAGCCA |  |
| qBm67-F | GGCGCTTCGATATTAGCCCA | qRT-PCR analysis of *Bm67* of BmNPV |
| qBm67-R | TTGAAACGTTCGCGGTTGAC |  |
| qDNA_pol-F | ATAATGAAGGTCCGTCGCCC | qRT-PCR analysis of *DNA pol* of BmNPV |
| qDNA_pol-R | GGCACAAAATCCGTGCCTTT |  |
| qlef4-F | TGATTCCACGATGGCGAACA | qRT-PCR analysis of *lef4* of BmNPV |
| qlef4-R | TGCGGGCCAAAATTTCATCG |  |
| qlef5-F | GTTTGACATAAAGCCGCCCA | qRT-PCR analysis of *lef5* of BmNPV |
| qlef5-R | GCGTACACCCAGTGTTGGTA |  |
| qlef7-F | CATCGTCACGTTGAATCGGC | qRT-PCR analysis of *lef7* of BmNPV |
| qlef7-R | ACGGTGTTACACAGCGATCC |  |
| qp12-F | CTGTTGGAACCGATGCAAGC | qRT-PCR analysis of *p12* of BmNPV |
| qp12-R | TGTCGTAAACGCTCCCCAAA |  |
| qp143-F | ACAGCGGAGAGTCCAACAAG | qRT-PCR analysis of *p143* of BmNPV |
| qp143-R | TACGACTCGCTGCACTGTTT |  |
| qgp41-F | GCACATCAACATGATCAACG | qRT-PCR of *gp41* for analysis of viral genome copies |
| qgp41-R | TAAACTCATGATTCGCGCTC |  |
| qrpl32-F | CAGGCGGTTCAAGGGTCAATAC | Normalization of the qRT-PCR data |
| qrpl32-R | TGCTGGGCTCTTTCCACGA |  |
| qrpl27-F | GCAATCACGAAGTGTTTAAGC | Normalization of viral gDNA copy number |
| qrpl27-R | AGCACTACTTTACCCGGCTTC |  |
| Vprobe1-F | CCGCTGTCGTAACCTTGGTCAA | Amplification of the fragments in the genome coordinates of 50k-95k nt for the synthesis of viral probes |
| Vprobe1-R | GTTGAAAGCCGGATCTCGTG |  |
| Vprobe2-F | CACGAGATCCGGCTTTCAAC |  |
| Vprobe2-R | ATTGCATAGATTCGTGGACG |  |
| Vprobe3-F | CGTCCACGAATCTATGCAAT |  |
| Vprobe3-R | GTGCGCTCCGTTTGCAAAAC |  |
| Vprobe4-F | TAAAATGTGTTCCCGTGCCG |  |
| Vprobe4-R | CCCGAGTACTTGCAAATCGA |  |
| Vprobe5-F | TCGATTTGCAAGTACTCGGG |  |
| Vprobe5-R | ATTCGAGCAAGAGCGATTCC |  |
| Vprobe6-F | GGAATCGCTCTTGCTCGAAT |  |
| Vprobe6-R | GATCCGACAACAATATACCC |  |
| Vprobe7-F | GGGTATATTGTTGTCGGATC |  |
| Vprobe7-R | GCATCACACAATCTTCCTGG |  |
| Vprobe8-F | TACACTGCCGACGAGTACAA |  |
| Vprobe8-R | ATGGAATGCCCGTTTCAGAT |  |
| Vprobe9-F | ATCTGAAACGGGCATTCCAT |  |
| Vprobe9-R | CGTGGAAGCGATTCATTCCG |  |
| Vprobe10-F | CGGAATGAATCGCTTCCACG |  |
| Vprobe10-R | ATGCTACTAGTAAATCAGTCATACCAAGGC |  |
| **siRNAs** | | |
| Name | Sequences (5′-3′) | Applications |
| siCtr-sense | UUCUCCGAACGUGUCACGUTT | Negative control of siEGFP |
| siCtr-antisense | ACGUGACACGUUCGGAGAATT |  |
| siEGFP-sense | GCAAGCUGACCCUGAAGUUTT | Gene silencing of EGFP |
| siEGFP-antisense | AACUUCAGGGUCAGCUUGCTT |  |
| **Probes** | | |
| Name | Sequences (5′-3′) | Applications |
| probe-siEGFP | AAGCAAGCTGACCCTGAAGTT | Detection of siEGFP |

**Supplementary Table 2.** Abbreviations are used in this manuscript

| AcMNPV | *Autographa californica* multiple nucleopolyhedrovirus |
| --- | --- |
| Ago2 | Argonaute-2 |
| BmNPV | *Bombyx mori* nucleopolyhedrovirus |
| BV | budded virion |
| CAT | chloramphenicol acetyltransferase |
| CrPV | cricket paralysis virus |
| Dcr-2 | Dicer-2 |
| DCV | *Drosophila* C virus |
| dsRNA | double stranded RNA |
| DXV | *Drosophila* X virus |
| EGFP | enhanced green fluorescent protein |
| FHV | Flock House virus |
| HaSNPV | *Helicoverpa armigera* single nucleopolyhedrovirus |
| hpi | hours post infection |
| IIV6 | invertebrate iridescent virus 6 |
| MCS | multiple cloning sites |
| MOI | multiplicity of infection |
| nt | nucleotides |
| ORF | open reading frame |
| polh | [polyhedrin](https://www.sciencedirect.com/topics/medicine-and-dentistry/polyhedrin) |
| qRT-PCR | Quantitative real-time PCR |
| RISC | RNA-induced silencing complex |
| RNAi | RNA interference |
| RPM | reads per million |
| siRNA | small interfering RNA |
| TCID_50_ | median tissue culture infective dose |
| vsiRNA | virus-derived small interfering RNA |
| VSR | viral suppressors of RNAi |
| WT | wild type |
